# Incidence and Persistence of Viral Shedding in COVID-19 Post-acute Patients With Negativized Pharyngeal Swab: A Systematic Review

**DOI:** 10.3389/fmed.2020.00562

**Published:** 2020-08-28

**Authors:** Giovanni Morone, Angela Palomba, Marco Iosa, Teodorico Caporaso, Domenico De Angelis, Vincenzo Venturiero, Anna Savo, Paola Coiro, Dario Carbone, Francesca Gimigliano, Giovanni Iolascon, Stefano Paolucci

**Affiliations:** ^1^Santa Lucia Foundation, Rome, Italy; ^2^Multidisciplinary Department of Medicine for Surgery and Orthodontics, University of Campania Luigi Vanvitelli, Naples, Italy; ^3^Department of Industrial Engineering, University of Naples Federico II, Naples, Italy; ^4^Department of Anesthesia and Intensive Care Therapy, Military General Hospital, Rome, Italy; ^5^Department of Mental and Physical Health and Preventive Medicine, University of Campania Luigi Vanvitelli, Naples, Italy

**Keywords:** viral shedding, COVID-19, SARS-CoV-2, stool, post-acute phase

## Abstract

After the global spread of a severe acute respiratory syndrome caused by a coronavirus (SARS-CoV-2), factors that influence viral diffusion have gained great attention. Human-to-human transmission mainly occurs through droplets, but viral RNA clearance in different biological fluids in coronavirus disease 2019 (COVID-19) remains unclear. We aimed to correlate the presence and the relevant temporal patterns of SARS-CoV-2 viral RNA in biological specimens (stool, urine, blood, and tears) of the transmission with clinical/epidemiological features in patients with COVID-19. We focused on the time window between the positivity of reverse transcriptase-polymerase chain reaction (RT-PCR) tests from different specimens. We used the Mantel–Cox log rank test to verify the differences in terms of viral shedding duration, while we employed the Mann–Whitney *U*-test for subgroup analysis. This review protocol was registered with PROSPERO number: CRD42020183629. We identified 147 studies; we included 55 (1,348 patients) for epidemiological analysis, of which we included 37 (364 patients) for statistical analysis. The most frequently used specimens other than respiratory tract swabs were stool samples (or anal/rectal swabs), with a positivity rate of 48.8%, followed by urine samples, with a positivity rate of 16.4%; blood samples showed a positivity rate of 17.5%. We found that fecal positivity duration (median 19 days) was significantly (*p* < 0.001) longer than respiratory tract positivity (median 14 days). Limited data are available about the other specimens. In conclusion, medical and social communities must pay close attention to negativization criteria for COVID-19, because patients could have longer alternative viral shedding.

## Introduction

At the end of the 2019, a novel coronavirus was isolated from patients with pneumonia in Hubei province, China; it was named the 2019 novel coronavirus (2019-nCoV), and the related severe acute respiratory syndrome was referred to as SARS-CoV-2 (1). On January 30, 2020, the World Health Organization (WHO) announced that the new emerging coronavirus pneumonia epidemic constituted a public health emergency of international concern (2). On March 11, 2020, due to the exponential increase in the number of reported cases and the high number of deaths (3), WHO's General Director announced that the novel coronavirus disease (COVID-19) may be defined as a pandemic.

The main sources of infection are SARS-CoV-2-infected patients, who produce a large quantity of the virus in the upper respiratory tract during a prodromal period and clinical manifestations. However, many factors play a crucial role in augmenting diffusion, such as the presence of asymptomatic carriers, the incubation period of the disease (usually ranging from 1 to 14 days, and even up to 24 days), and the mild clinical symptoms during the first disease period, with infected subjects still having an active life (4, 5).

Our understanding of SARS-CoV-2 human-to-human transmission is still evolving; currently, we know that it mainly occurs through air droplets. However, feces may be another potential route of transmission (6). Nosocomial transmission is a severe problem, given the susceptible condition of inpatients, so any action should be taken to minimize the risk of transmission. Notably, there is no indication regarding the danger of biological fluids from a patient with a negative pharyngeal swab. This could become a major problem if he or she is admitted to a post-acute hospital ward or to any sanitary structure with lower healthcare assistance or when he or she is discharged into the community, as demonstrated by a recent review on gastrointestinal symptoms (7). Subjects with positive viral RNA excretion need to be isolated; however, the persistence and clearance of viral RNA in different biological fluids remains unclear. Thus, as the clearance of viral RNA from patients' stool is delayed compared with that from oropharyngeal swabs, it is important to detect the viral RNA in feces during the convalescence phase to provide guidance to patients about contact limitations and even to manage drug administration (i.e., avoiding immunosuppressant drugs such as glucocorticoids).

In this context, our study, inspired by the needs expressed by physicians in post-acute settings, aimed to systematically review the existing data on novel coronavirus viral shedding. We reviewed, referring to the recommended diagnostic criteria: (i) the incidence of viral RNA in biological specimens (urine, stool, blood, and tears); (ii) the persistence of viral shedding and the correlation between the presence of viral RNA in the respiratory tract and in feces; and (iii) the correlation between persistent viral shedding in the post-acute phase with disease severity.

## Methods

### Search Strategy and Selection Criteria

For our systematic review and meta-analysis, we followed PRISMA guidelines (8). We searched for data on confirmed COVID-19 patients' viral shedding reported in any kind of study (case report/series, cohort studies, case-control studies, or randomized control trials) with available data in English, published until May 5, 2020. Two authors (G.M. and A.P.) independently and synchronously searched PubMed, EMBASE, and Web of Science up to May 5, 2020, in order to identify all studies documenting modalities of SARS-CoV-2 viral shedding in patients with a confirmed diagnosis of COVID-19.

The search terms were “2019-nCoV,” “SARS-CoV-2,” “novel coronavirus,” or “COVID-19” combined with “viral shedding” and/or “feces,” “stool,” “feces,” “urine,” “blood,” or “tears.” We found additional studies by carefully searching the reference lists of the identified works. Titles and abstracts were screened, and two authors (G.M. and A.P.) independently reviewed full-text papers. Exclusion criteria were studies not written in English, not reporting specimens other than respiratory tract swabs, duplicates, or not matching the inclusion criteria and/or the topic of the review (for this last criterion, in case of disagreement between the two above authors, an independent reviewer stepped in, namely D.D.).

We obtained data about the sites of studies, sample sizes, patient demographics, analyzed clinical samples, disease duration, and viral shedding duration through different routes. We then focused on the time window between the positivity of reverse transcriptase-polymerase chain reaction (RT-PCR) tests from different specimens. In particular, we considered the duration of sample positivity for SARS-CoV-2 from the onset of symptoms or, for asymptomatic patients, from the first positive result until the last available positive testing. We considered respiratory samples (throat swabs, nasopharyngeal swabs, oral swabs, sputum, or saliva) to be a hallmark of COVID-19 diagnosis, while we compared the other specimens' duration of positivity to the respiratory one. We collected specific data about single patients when available. When possible, we asked corresponding authors for missing data in order to collect wider information. When we could not obtain single patient data, we took pooled data.

### Data Analysis

M.I. performed all statistical analyses using Statistical Package for Social Science (SPSS) 25.0. Continuous variables are expressed as median (interquartile range) or mean (± standard deviation), according to their normality test results (verified through the Shapiro–Wilk test). In order to overcome the possible heterogeneity within and between studies, M.I. performed the analyses on a pooled database containing data from each patient enrolled in the studies that provided single subject data and not using aggregated measures. M.I. used the Mantel–Cox log rank test to verify the differences in terms of viral shedding duration, while M.I. used the Mann–Whitney *U*-test for subgroup analysis. Moreover, we assessed the quality of the selected studies using the Newcastle–Ottawa Scale (9). We registered our review on PROSPERO (registration number: CRD42020183629).

## Results

**Figure 1:**
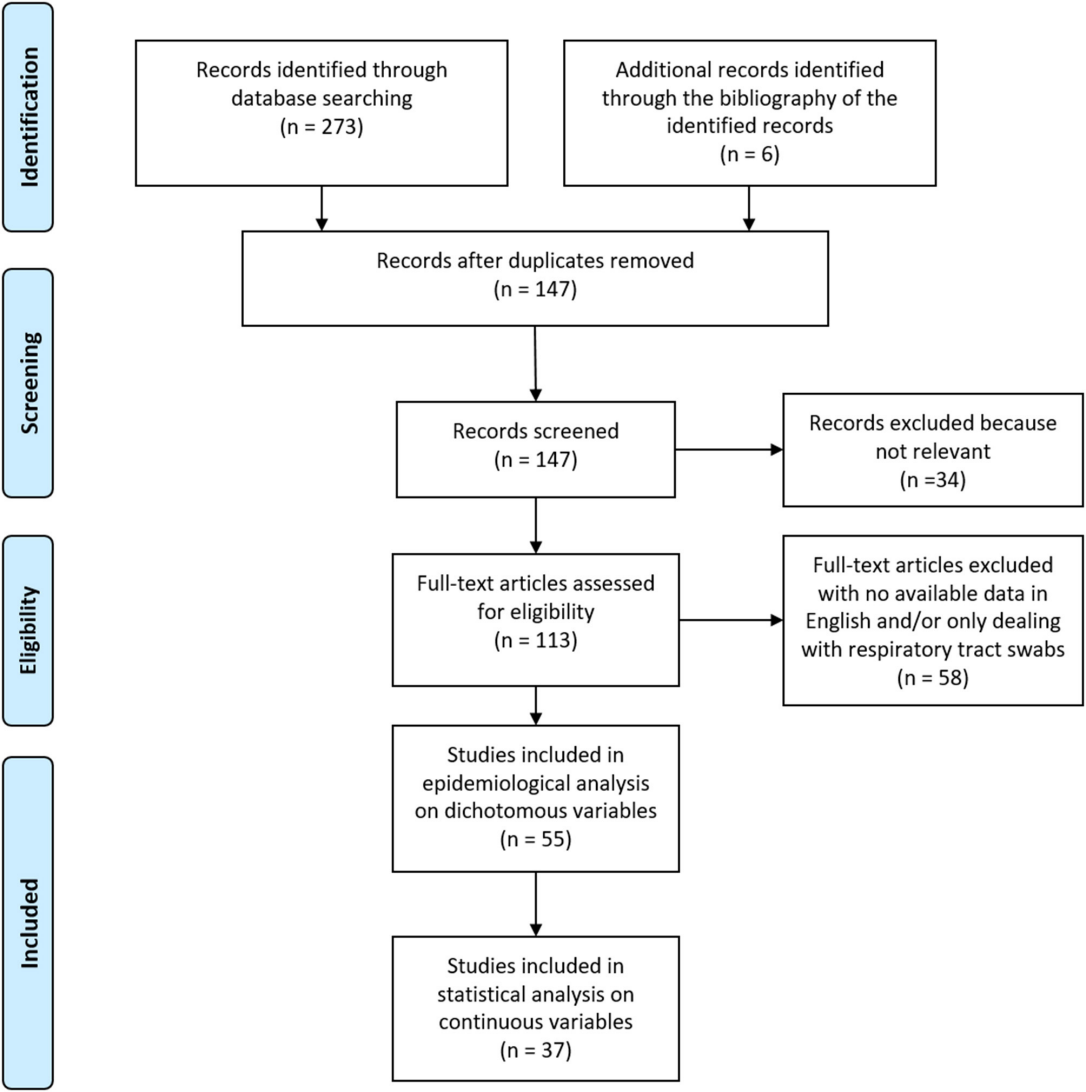
Flow diagram of the search results, according to the PRISMA recommendations.

The results of our search are shown in the PRISMA flow-chart depicted in Figure 1. After removal of duplicates and documents assessed as not eligible for our purposes, we found 113 papers. Of these, we included 55 studies in the present review for epidemiological analysis on group data and dichotomous variables; 37 of these reported continuous values and could be included in our quantitative analysis on single patients' data.

The detailed data of the 55 selected studies are available in Table 1. All the selected articles used RT-PCR for viral RNA detection (10–64). A few of them (32, 41, 42, 63) added viral cultures, viral isolation, or next generation sequencing (NGS). The total number of patients was 1,348 (1–132 for each article), with an age range from 17 days to 96 years. Of 1,219 patients for whom we found information about gender, 593 were female (48.6%). Most of the studies (78.2%) were conducted in China, while the others were from Asia (two from Korea, two from Singapore, one from Taiwan, and one from Lebanon), Europe (two from Italy, one from France, and one from Germany), and the United States of America (two). Almost all studies (52 out of 55) were assessed as high quality, showing Newcastle–Ottawa Scale scores ≥7 (9).

**Table 1:** Detailed information of the included studies (8–62).

	**First author**	**Country**	**Sample**	**Method**	**Total patient number**	**Specimen positivity**	**Age range (years)**	**Sex**	**NOS**
1	Wu	China	R, F	RT-PCR	74	41 F+	40–52	35 Fem 39 Mal	9
2	Zhang Y	China	R, F	RT-PCR	15	5 F+	37 (10–73)	7 Fem 8 Mal	8
3	Xu	China	R, F	RT-PCR	10	8 F+	0.2–15.7	4 Fem 6 Mal	8
4	Xing YH	China	R, F	RT-PCR	3	3 F+	1.5–6	1 Fem 2 Mal	8
5	Chen	China	R, F, U	RT-PCR	42	28 F+	51 (42.75–62)	24 Fem 18 Mal	9
6	Lo	China	R, F, U	RT-PCR	10	10 F+	54 (27–64)	7 Fem 3 Mal	9
7	Nicastri	Italy	R, F, U, O	RT-PCR	1	1 F+	29	1 Mal	7
8	Young	Singapore	R, F, Bl, U	RT-PCR	8	4 F+	n. a.	n. a.	7
9	Holshue	USA	R, F, Bl	RT-PCR	1	1 F+	35	1 Mal	7
10	Cai	China	R, F, Bl, U	RT-PCR	6	6 F+	0.3–10.9	4 Fem 2 Mal	8
11	Zhang JC	China	R, F	RT-PCR	14	5 F+	18–87	7 Fem 7 Mal	8
12	Zeng L	China	R, F	RT-PCR	1	1 F+	0.46	1 Mal	7
13	Yang Z	China	R, F	RT-PCR	3	3 F+	25–62	1 Fem 2 Mal	7
14	Xiao F	China	R, F	RT-PCR	73	39 F+	43 (0.83–7)	32 Fem 41 Mal	7
15	Zheng	China	R, F, Bl, U	RT-PCR	96	55 F+, 39 Bl+, 1 U+	55 (44.3–64.8)	38 Fem 58 Mal	8
16	Pan	China	R, F, U	RT-PCR	11	–	n. a.	n. a.	7
17	Cheng	Taiwan	R, F, U	RT-PCR	1	–	55	1 Fem	7
18	Kim	Korea	R, F, Bl, U	RT-PCR	2	–	35–55	1 Fem 1 Mal	8
19	Qian	China	R, F	RT-PCR	1	1 F+	47	1 Mal	7
20	Xing	China	R, F	RT-PCR	1	–	40	1 Mal	7
21	Tang	China	R, F	RT-PCR	1	1 F+	10	1 Mal	7
22	Tan	China	R, F, Bl	RT-PCR	4	3 F+	3.5–9	3 Fem 1 Mal	8
23	Mansour	Lebanon	R, F, U	RT-PCR/ cultures	1	–	1.41	1 Fem	7
24	Chen	China	R, F	RT-PCR	22	12 F+	2–64	8 Fem 14 Mal	9
25	Han	Korea	R, F, Bl, U	RT-PCR	2	1 F+ Bl+ U+ Sal+, 1 F+	55	2 Fem	7
26	Zhang T	China	R, F	RT-PCR	3	3 F+	6–9	3 Mal	8
27	Yuang	China	R, F	RT-PCR	6	6 F+	36–71	4 Fem 2 Mal	8
28	Liu	China	R, F	RT-PCR	4	4 F+	8–46	2 Fem 2 Mal	8
29	Li J	China	R, F, Bl, U, Vag, Mil	RT-PCR	13	3 F+	1–73	7 Fem 6 Mal	8
30	Jiang	China	R, F	RT-PCR	1	1 F+	8	1 Fem	7
31	Paoli	Italy	R, U, Sp	RT-PCR	1	–	31	1 Mal	7
32	Seah	Singapore	R, O	RT-PCR/viral isolation	17	–	20–75	6 Fem 11 Mal	7
33	Lescure	France	R, F, Bl, U, O	RT-PCR/viral isolation	5	2 F+, 1 Bl+	30–80	2 Fem 3 Mal	9
34	Wölfel	Germany	R, F, Bl, U	RT-PCR	16	8 F	35 (2–58)	4 Fem 12 Mal	7
35	Kujawski	USA	R, F, Bl, U	RT-PCR	10	6 F+, 1 F+ Bl+	53 (21–68)	3 Fem 7 Mal	9
36	Su	China	R, F	RT-PCR	4	4 F+	0.9–3.6	2 Fem 2 Mal	8
37	Sun	China	R, U	RT-PCR	1	1 U+	72	1 Mal	7
38	Cheung	China	R, F	RT-PCR	59	9 F+	22–96	32 Fem 27 Mal	6
39	Zhang W	China	R, F, Bl	RT-PCR	16	10 F+	n.a.	n.a.	7
40	Ling	China	R, F, U	RT-PCR	66	66 F+ (4/58 U+)	34–62	38 Fem 28 Mal	7
41	Lei	China	R, F	RT-PCR	7	4 F+	n.a.	n.a.	6
42	Wu	China	R, F, Bl	RT-PCR	132	36 F+, 4 Bl+	66.7 ± 9.1	60 F 72 M	7
43	Ma	China	R, F	RT-PCR	8	5 F+	0.9–39	6Fem 2 Mal	7
44	Fang	China	R, F, Bl, O	RT-PCR	32	23 Bl+; 5 O+	41 (34–54)	16 Fem 16 Mal	7
45	Wei	China	R, F	RT-PCR	84	28 F+	37 (24–74)	56 Fem 28 Mal	7
46	Qian GQ	China	R, F	RT-PCR	91	2 F+	5–96	54 Fem 37 Mal	8
47	Peng	China	R, F, Bl, U	RT-PCR	7	1 F+, 1 F+ Bl+1 Bl+, 1 U+	27–49	4 Fem 3 Mal	7
48	Yun	China	R, F, Bl, O	RT-PCR	32	8 F+	50 (37–66)	17 Fem 15 Mal	7
49	Wang	China	R, U	RT-PCR	116	53 U+	54 (38-69)	49 Fem 67 Mal	7
50	Yu	China	R, Bl, U	RT-PCR/dd-PCR	76	4 Bl- 14 U-	40 (32–63)	38 Fem 38 Mal	8
51	Lin	China	R, F, Biop	RT-PCR	65	31 F+, 3/6 Biop+	n.a.	n.a.	7
52	Wang	China	R, F, Sew	RT-PCR	2	1 F+, Sew+	n.a.	n.a.	6
53	Xie	China	R, F, Bl, U	RT-PCR	9	8 F+	18–62	5 Fem 4 Mal	7
54	Huang J	China	R, Bl	RT-PCR/NGS	41	6 Bl+	49 (41–58)	11 Fem 30 Mal	8
55	Wang W	China	R, F, Bl	RT-PCR	20	6 F+, 2 Bl+	n.a.	n.a.	7

*NOS, Newcastle—Ottawa Quality Assessment Scale; R, respiratory tract swabs; F, fecal samples; U, urine samples; Bl, blood; O, ocular samples; Vag, vaginal samples; Mil, Breast Milk; Biop, biopsies; Sew, sewage; +, number of positive samples; Fem, female; Mal, male; n. a., not available*.

**Figure 2:**
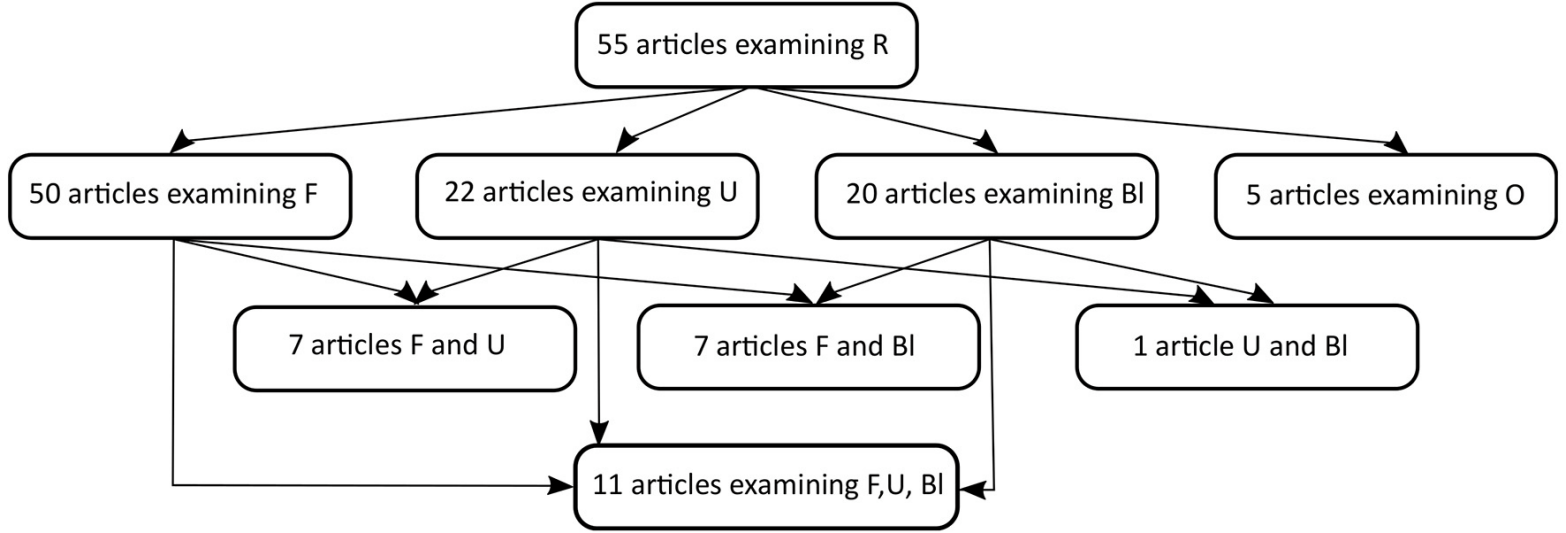
Flow diagram of the specimens considered in the 55 selected articles (R, respiratory tract; F, feces; U, urine; Bl, Blood; O, ocular samples). We want to clarify that two of the F and Bl, one of the F, U, Bl and one of the F and U even consider ocular samples.

As shown in Figure 2, the most frequently used specimens other than respiratory tract swabs were stool samples (or anal/rectal swabs). Indeed, 50 articles examined fecal samples, with a positivity rate of 48.8% (490 out of 1,005 patients). Moreover, 22 articles examined urine samples, with a positivity rate of 16.4% (60 out of 366 patients), while blood samples showed in 20 articles a positivity rate of 17.5% (80 out of 456 patients). Finally, five articles considered ocular samples (tears or conjunctival swabs), with a positivity rate of 7.7% (5/65 patients). However, most of these studies did not report the duration data of each tested patient. One study (16) examined the semen of only one patient, with a negative result, while another study (38) looked for coronavirus RNA in the breast milk of a breastfeeding woman, also with a negative result. Another study (60) added the virus search on gastrointestinal tract biopsies (with three positive results out of six biopsies). Wang and colleagues (62) analyzed sewage and identified SARS-CoV-2 RNA.

**Figure 3:**
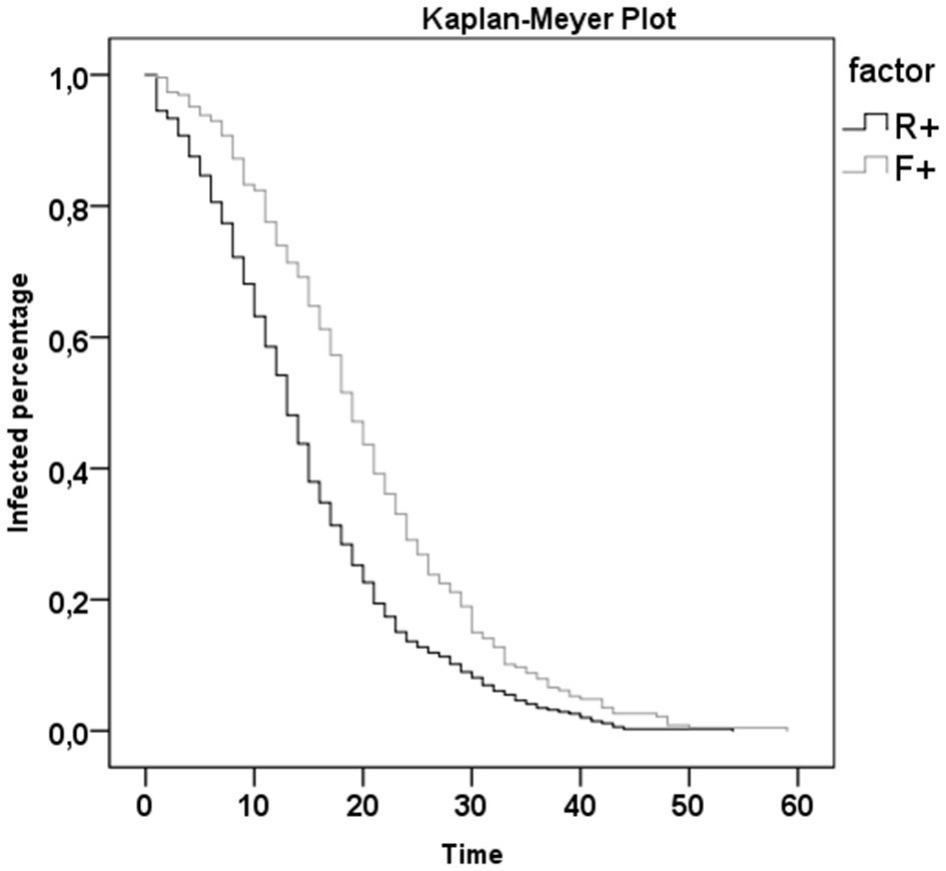
Kaplan-Meyer Plot of R+ (respiratory tract swab positivity) and F+ (fecal sample positivity) duration.

From 37 studies (including 364 patients) reporting the duration of both R+ (respiratory tract swab positivity) and F+ (fecal sample positivity) for each patient (9–45), we pooled data for statistical analysis. Although these studies included 364 patients, R+ and F+ duration data were only available for 215 individuals, plus 11 patients for whom only the difference between F+ and R+ had been reported. The median R+ duration was 14 days [interquartile range (IQR) 12 days], whereas that of F+ was 19 days (IQR 14 days). The Shapiro–Wilk test highlighted that both R+ and F+ were not normally distributed (*p* < 0.001). For this reason, we used the Wilcoxon test to compare the lengths of positivity; there was a statistically significant difference (*p* < 0.001, *n* = 215). There was a significant correlation between the duration of R+ and F+ (Spearman correlation coefficient R = 0.507, *p* < 0.001). The Mantel–Cox log rank showed a statistically significant difference between F+ and R+ trends (χ^2^ = 31.6, *p* < 0.001; Figure 3). Of the 226 patients with both R+ and F+, 27 patients (11.9%) had the same duration for both routes of viral shedding, 55 (24.3%) had a longer R+ duration, and the remaining 144 (63.7%) showed a longer F+ duration.

**Figure 4:**
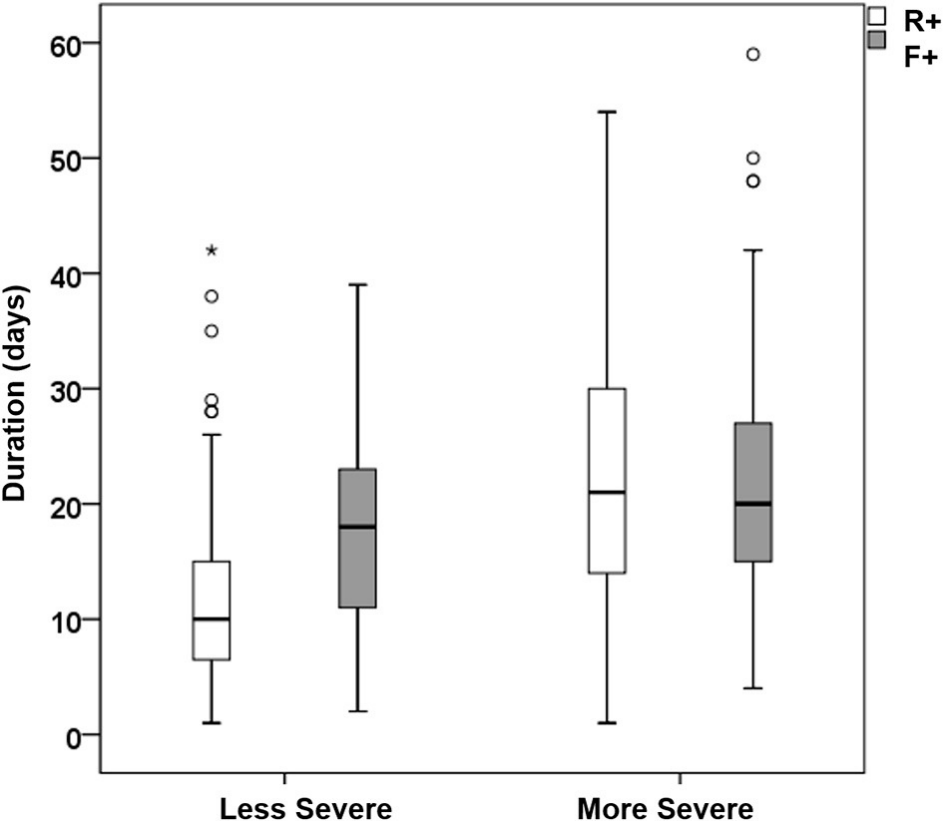
Box-whiskers plot with median and quartile values for the duration of R+ (white boxes) and F+ (gray boxes) of less (left) and more (right) severe patients (circles and stars indicate patients out of the 95% confidence interval).

Moreover, there were statistically significant differences between severe and not severe [as defined by the American Thoracic Society and Infectious Disease Society of America guidelines for community acquired pneumonia (65)] patients in terms of R+ duration (*p* < 0.001, Mann–Whitney *U*-test, *n* = 309), F+ duration (*p* = 0.010, *n* = 184), and their difference (*p* < 0.001, *n* = 182). Interestingly, for the most severe subjects, R+ and F+ durations were not statistically different from each other (*p* = 0.496, Wilcoxon test, *n* = 69), whereas for less severely affected patients, there was a statistically significant difference (*p* < 0.001, *n* = 112; Figure 4).

There were age data available for 105 subjects, 41 of whom were children (age <18 years old), 38 of whom showed mild symptoms (according to the literature). For this reason, we compared the data of these 38 children with those of adults with mild symptoms for whom age data were available (*n* = 48). We did not find significant age-related differences in terms of R+ (median 8 days, IQR 9 days in children vs. median 10 days, IQR 11 days in adults; *p* = 0.121) or F+ (median 22 days, IQR 12 days in children vs. median 18 days, IQR 12 days in adults; *p* = 0.058). However, the difference between F+ and R+ was significantly longer in children than in adults (median 12 days, IQR 12 days in children vs. median 5 days, IQR 11 days in adults; *p* = 0.001).

Statistical analysis about the duration of other specimen positivity (blood, urine, and ocular samples) was not possible, due to the reduced sample size of available data. Indeed, single patient data about blood sample positivity were reported only in three studies (24, 34, 42), and urinary sample positivity duration was available for one single case study (34). There were no available data on other specimens for single patients.

Finally, digestive symptoms were available for 42 patients, but all from the same study (14). For this reason, it was not possible to perform a meta-analysis on these symptoms.

## Discussion

The available data confirm the presence of viral RNA in several biological specimens (stool, urine, blood, and tears), but with very different positivity rates. Our results confirm concerns initially identified by Zhang and colleagues in their pioneering work (48). These concerns are related to modalities of dealing with people considered recovered after COVID-19 infection, without considering the persistent viral shedding in their biological specimens other than those collected in the respiratory tract. Not keeping them isolated or not taking the appropriate precautions could markedly increase the risk for virus spreading during the post-acute phase. Indeed, the present work confirms, on a wider basis than previous studies (364 patients), the significant prolonged viral shedding through feces. Although our aim was to also analyze other specimens, most of the analyzed studies only reported respiratory tract and fecal data. Our results revealed that the prolonged positivity of viral RNA excretion was statistically significant, particularly in patients with less severe disease, although digestive symptoms had only been anecdotally reported in previous review studies (14). This outcome may depend on the inclusion/exclusion search criteria of our review. Other reviews have focused on gastrointestinal symptoms and reported a higher prevalence in more severe patients (46, 65). Our findings suggest the importance of screening the viral positivity of patients' stool even after negative results of their respiratory tract swabs. Therefore, prolonging the contact precautions both at home or in the post-acute environment for all post-COVID-19 patients seems to be advisable. The Kaplan–Meyer plot (Figure 3) would suggest prolonging the precautions for about 10 days. Moreover, as suggested by Yeo et al. (66), it is important to clarify the possibility of fecal-oral transmission for SARS-CoV-2, as already confirmed for other coronaviruses (67). In addition, a recent review by Cheung (47) investigated the correlation between fecal viral shedding duration and enteric symptoms. Finally, we analyzed the correlation between viral RNA excretion in feces and the disease severity. The longer duration of viral shedding in feces was statistically significant for less affected patients, and especially for children, a population in which the severity of COVID-19 was lower, as has been widely reported in literature.

The main limitation of this work is related to the fact that most of the studies detected viral RNA and not live viral shedding. So far, the exact correlation between RNA viral shedding and infectious viral shedding is not known, although live SAR-CoV-2 viruses have been isolated in different specimens including stool (68). We identified four other limitations: (1) despite our aim to analyze viral shedding in specimens other than respiratory swabs, most of the data were limited to feces; (2) specimens collected in different areas of the same body tract are considered a single type of sample; (3) we analyzed all data available in the publications about positivity rates and viral RNA shedding duration, but we must consider possible biases in the previous publications, for which only part of the data had been published by the authors (publication bias); and (4) the duration of infection might depend on the criteria related to the diagnosis of infection and to those for defining the negativization of a patient (with one or two consecutive negative tests) potentially related to different tracts (nasal or fecal swab tests) and different symptoms (respiratory or digestive).

## Conclusions

In conclusion, on the basis of our results, medical and social communities must pay close attention to patients who present COVID-19 with mild or no symptoms, because our results suggest they could represent individuals with longer alternative viral shedding, even after a negativized pharyngeal swab. Therefore, appropriate management of the patient flow between an intensive care unit (ICU) and post-ICU departments (i.e., post-acute units) should be carefully considered by implementing risk management that is also related to alternative viral shedding.

## Data Availability Statement

All datasets generated for this study are included in the article/supplementary material.

## Author Contributions

GM and AP performed the literature search and wrote the first draft of the report, with input from DC. TC assessed the quality of the selected studies. DD, VV, AS, PC, FG, GI, and SP reviewed the draft and expanded the clinical implications. MI performed the statistical analysis and had full access to all the data in the study. All authors contributed to the article and approved the submitted version.

## Conflict of Interest

The authors declare that the research was conducted in the absence of any commercial or financial relationships that could be construed as a potential conflict of interest.
